# Supplementary material for: Biasing Influence of ‘Mental Shortcuts’ on Diagnostic Decision-Making: Radiologists Can Overlook Breast Cancer in Mammograms When Prior Diagnostic Information Is Available
Source: Diagnostics (Basel). 2022 Jan 4;12(1):105. doi: 10.3390/diagnostics12010105 (PMC8774943; doi:10.3390/diagnostics12010105)
Supplement: Supplementary file 1 [file diagnostics-12-00105-s001.zip › diagnostics-1468280-supplementary.pdf]

diagnostics

MDPI

Brief Report

Biasing Influence of 'Mental Shortcuts' on Diagnostic Decision-Making: Radiologists Can Overlook Breast Cancer in Mammograms When Prior Diagnostic Information is Available

Fallon Branch, Isabella Santana and Jay Hegdé

Supplementary Materials

Supplementary Table S1. Post hoc general linear modeling (GLM) of the radiologists' responses in Study 1. The GLM estimated the contributions of the various explanatory variables to the response variable (i.e., final estimates β of radiologists). Qualitatively similar results (not shown) were obtained when the subjects' initial estimate α (row 1) was replaced by the purported prior estimate ψ as one of the explanatory variables. Similarly, qualitatively similar results (not shown) were obtained when the stimulus duration *t* (row 3) was replaced by reaction time (not shown) as one of the explanatory variables. The above GLM results were confirmed using standard model selection procedures that screened the various predictor variables as to whether they significantly accounted for the final estimates[24] (not shown), which indeed retained the initial estimates α as the sole explanatory variable ($F(1,142) = 25.54$; $r^2 = 0.15$, $p = 1.31 \times 10^{-6}$).

Row #	Explanatory Variable	Estimated Coefficient	Standard Error	*t* Value	*p* Value
1	Subjects' initial estimate α	0.43	0.09	4.96	2.02×10^{-6}
2	Cancer status of the mammogram (-ve *vs*. +ve for cancer) θ	-8.09×10^{-2}	4.65	−0.02	0.99
3	Stimulus duration *t*	-6.59×10^{-5}	1.1×10^{-4}	−0.60	0.55
4	Radiological specialty of the subject	8.15	6.85	1.19	0.24
5	Length of radiological experience of the subject	−0.46	0.29	−1.55	0.12

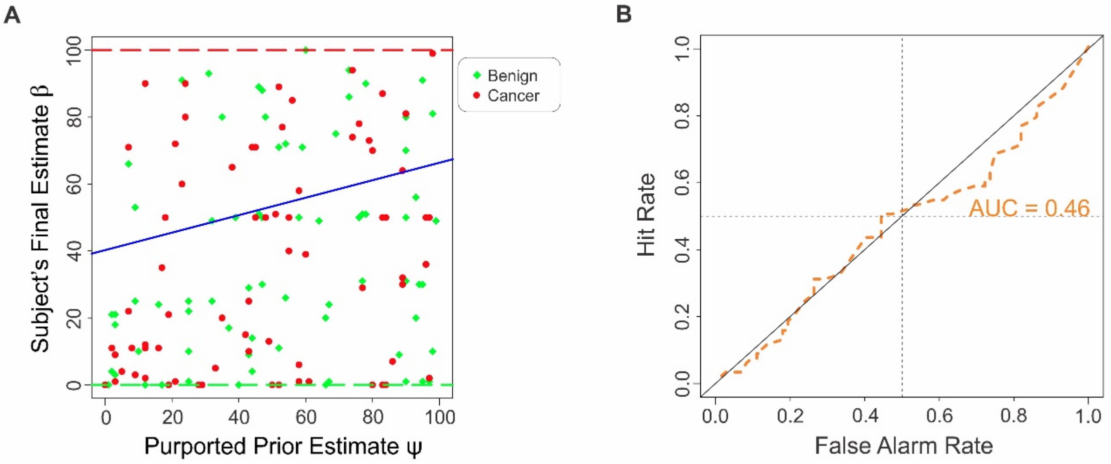

Supplementary Figure S1. Additional analyses of the subjects' responses in Study 1. **A**, Radiologists' reported final estimate β as a function of the purported prior estimate ψ. The *red* and *green dashed lines* denote the expected responses for mammograms positive and negative for cancer, respectively. **B**, Receiver operating characteristic (ROC) analysis of the subjects' performance in cancer detection. The ROC curve (*dashed brown* line) and the area under the ROC curve (AUC; *brown* type) are shown. The *diagonal* represents chance performance (AUC = 0.5). See text for details.

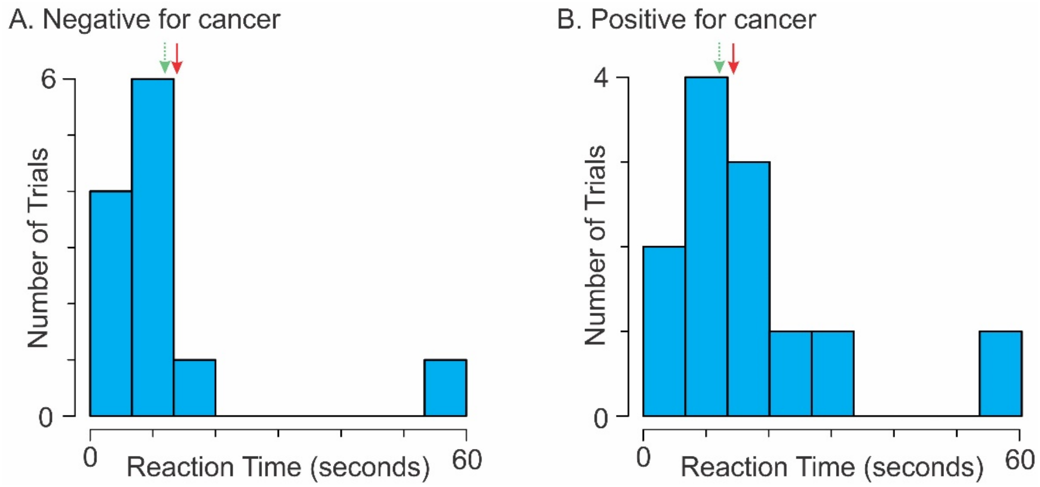

Supplementary Figure S2. Reaction times of the subjects in Study 2. The subjects viewed the stimuli for much shorter periods than they were allowed to in Study 1. This figure shows the reaction times for the trials in which the nominal stimulus duration was 60 s (i.e., the stimulus could be viewed for up to 60 s). The *solid red* and *dashed green arrows* indicate the mean and median viewing durations, respectively. Viewing durations did not significantly differ based on whether the mammogram was actually positive for cancer for the nominal stimulus duration of the 60 s (panel **A** *vs.* panel **B** in this figure; two-tailed *t*test, $t = -0.58$, $df = 139.11$, $p = 0.561$) or across all stimulus durations (not shown).

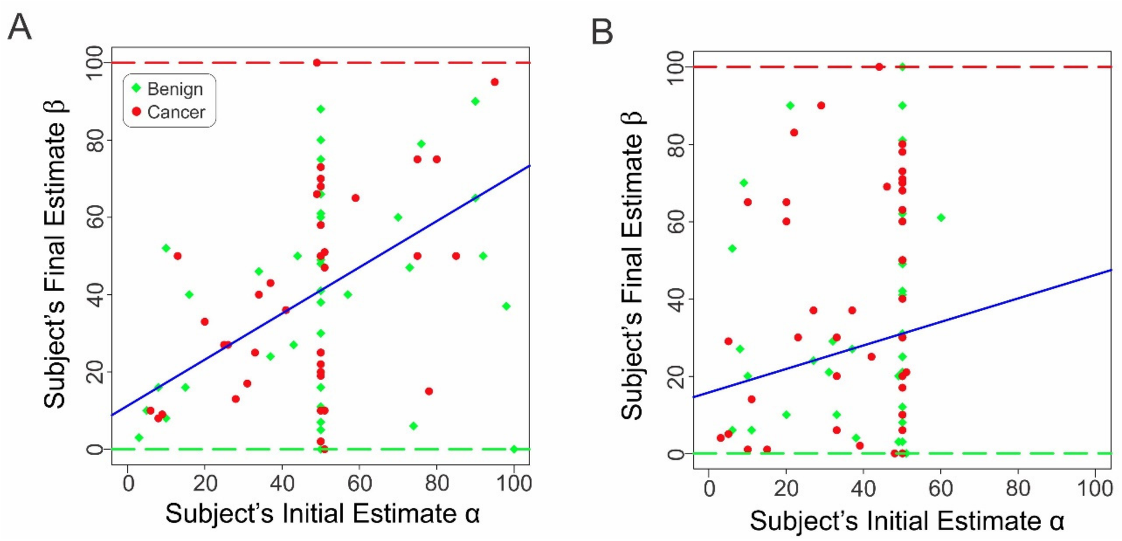

Supplementary Figure S3. Subjects' final estimates in Study 2 with or without anchoring information. **A**, Subjects' reported final estimate β as a function of their initial estimate α without anchoring information. **B**, Final estimate β as a function of initial estimate α with anchoring information. See text for details.
